# New techniques and methods for prevention and treatment of symptomatic traumatic neuroma: A systematic review

**DOI:** 10.3389/fneur.2023.1086806

**Published:** 2023-02-16

**Authors:** Liping Zhou, Tong Huo, Wenmin Zhang, Na Han, Yongqiang Wen, Peixun Zhang

**Affiliations:** ^1^Key Laboratory of Trauma and Neural Regeneration, Department of Orthopaedics and Trauma, Peking University People's Hospital, Peking University, Beijing, China; ^2^Beijing Key Laboratory for Bioengineering and Sensing Technology, Daxing Research Institute, School of Chemistry and Biological Engineering, University of Science and Technology Beijing, Beijing, China

**Keywords:** traumatic neuromas, complex symptoms, functional biomaterial therapy, stem cell technology, human machine interface

## Abstract

Generally, axons located at the central end of the nerve system will sprout after injury. Once these sprouts cannot reach the distal end of the severed nerve, they will form a traumatic neuroma. Traumatic neuromas bring a series of complex symptoms to patients, such as neuropathic pain, skin abnormalities, skeletal abnormalities, hearing loss, and visceral damage. To date, the most promising and practical clinical treatments are drug induction and surgery, but both have their limitations. Therefore, it will be the mainstream trend to explore new methods to prevent and treat traumatic neuroma by regulating and remodeling the microenvironment of nerve injury. This work first summarized the pathogenesis of traumatic neuroma. Additionally, the standard methods of prevention and treatment on traumatic neuroma were analyzed. We focused on three essential parts of advanced functional biomaterial therapy, stem cell therapy, and human-computer interface therapy to provide the availability and value of preventing and treating a traumatic neuroma. Finally, the revolutionary development of the prevention and treatment on traumatic neuroma has been prospected. How to transform the existing advanced functional materials, stem cells, and artificial intelligence robots into clinical practical technical means as soon as possible for high-quality nerve repair and prevention of neuroma was further discussed.

## 1. Introduction

The traumatic neuroma is usually caused by the random arrangement of nerve branches at different sizes after nerve injury (1). Macroscopically, the neuroma usually presents as a white oval mass surrounded by dense fibers, low blood vessel density, and nerve connections (2). Its size generally does not exceed 2 cm (2–4). According to the pathophysiological mechanism, the traumatic neuroma could be divided into two categories: terminal neuroma (i.e., “terminal” or “stump”) and discontinuous neuroma (4, 5). Not all neuromas are dangerous and problematic, but symptomatic neuromas could cause sensory disorders and persistent pain (6), and ultimately lead to a significant decline in patients' quality of life. It was reported that the incidence of painful neuromas in amputees has been as high as 50–80% (3). The International Association for Pain Research (IASP) (7) defines neuropathic pain as pain caused by diseases or lesions of the somatosensory nervous system (8). The arguments that mediate the pain mechanism of a neuroma include peripheral and central sensitization (9), expression of nerve growth factor (10), α-smooth muscle actin (α-SAM) (11), the change of neuroma fiber structure (2), connection between fibers, destruction of nerve function, and adrenaline sensitivity, etc. To avoid pain caused by neuroma, the injured or severed nerves should be treated with high quality.

The clinical treatment of neuroma was mainly divided into conservative treatment and surgical treatment (3). Among them, traditional conservative treatment specifically includes early prevention, intervention, and non-surgical treatment, such as anesthesia steroid injection, opioids, transcutaneous electrical nerve stimulation, and other methods (3). Once the symptoms and pain become severe, the neuroma needs surgical treatment, which includes epineural grafts, suture ligature, transposition into vein graft, muscle, bone, and traction neurectomy (12–14). The specific differences and connections between the two treatment modes were shown in Table 1. However, there are limitations to both treatments mentioned above. For example, up to 42% of patients may have persistent symptoms and undergo additional intervention (4). Therefore, exploring and developing new technologies are mainstream trend in the future prevention and treatment on a traumatic neuroma.

**Table 1:** Two different clinical treatments for neuroma.

**Treatment methods**			**Advantages and disadvantages**
Conservative treatment	Early prevention and intervention		Avoid pulling and compressing the nerve; Cut the nerve away from the stump quickly or carbonize the nerve with electric knife, and give professional rehabilitation massage after operation
	Nonoperative treatment	Bioactive substances (15);	Reduce the pain intensity and sensitivity of neuroma; Early patients or adjuvant therapy for patients with painful neuroma after surgery
		Professional rehabilitation massage	
		Radiofrequency ablation	
		Electrical stimulation	
Surgical treatment	Resection of simple neuroma		Simple operation; Postoperative recurrence or reoperation;
	Closure of nerve stump		Repair of injured nerve with artificial catheter; Simple operation; Reduce pain;
	Nerve stump dredging	Method of embedding nerve stump into muscle	Reduce pain and tenderness; Provide additional cushioning and protection;
		Embedding nerve stump into blood vessel	
		Intramedullary implantation of nerve stump	
	Reconstruct neural continuity	End to end neurorrhaphy	Less recurrence; Reduce pain; Allogeneic transplantation; Accurate operation; Long cycle of nerve recovery
		End to side neurorrhaphy	
		Nerve transplantation	
	Reinnervation	Targeted muscle reinnervation	Appropriate free muscle size; Transplanted muscle with vascular reconstruction
		Regenerative peripheral nerve interface	

Supramolecular biomaterials have developed rapidly in biomedical treatment due to their incomparable characteristics. The complexity of biology, pathology, and related biomedical issues requires that supramolecular biomaterials should be practical and designable. The designability and modifiability of supramolecular biomaterials (16) endow them with many advantages, such as responsiveness, reversibility, adjustability, bionics, modularity, predictability, and adaptability, which provides unlimited possibilities and opportunities to solve challenging biomedical problems (17). Therefore, the combination of advanced functional biomaterials and pathology provides a favorable guarantee for treating and preventing traumatic neuroma.

This review has focused on the research progress in the cross field of supramolecular biomaterials, stem cells, human-computer interface, and neuroma medicine in recent years. We hope this can make contributions to the treatment of traumatic neuroma. In addition, based on many excellent basic research, the effect of various new technologies and surgical treatment schemes should be strictly compared to prevent and treat neuroma in the future.

## 2. Difficulties and challenges in nerve repair

**Figure 1:**
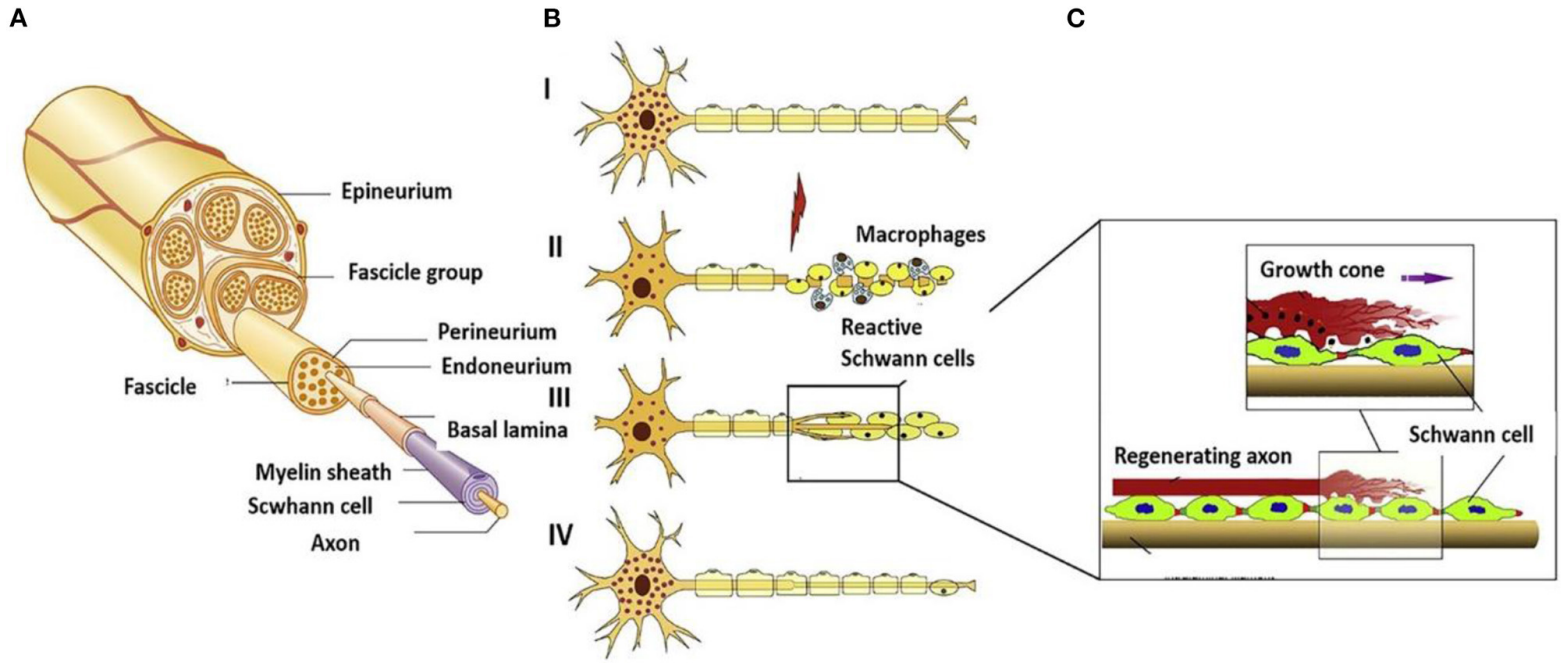
**(A)** Schematic diagram of peripheral nerves reprinted (adapted) with permission from (19), Copyright (2014) BioMed Research International. **(B)** Schematic diagram of disorder and regeneration after nerve damage reprinted (adapted) with permission from (20), Copyright (2012) Elsevier. **(C)** Schematic diagram of SCs surrounding the nerve axis reprinted (adapted) with permission from (21), Copyright (2014) Elsevier.

The occurrence of neuroma is actually a kind of transitional repair or body disorder in the process of nerve repair. The essence of improving or inhibiting the occurrence of neuroma is based on the research of biological mechanisms. Therefore, how to prevent the formation of neuromas requires profound understanding of neuroanatomy and neurobiological mechanisms after injury (18). As shown in Figure 1A, the central nervous system was composed of sensory and motor neurons (19). Their cytoplasm could spontaneously extend and gradually form axons, which were used to transmit signals to corresponding organs. Anatomically speaking, the nerve intima protects the axons to form nerve bundles. The adventitia gathers several nerve bundles together. In addition, there are a large number of capillary micro nerves with delicate network structures in the inner layer to provide energy. Once the nerve is injured (Figure 1B), different levels of the injured site may lead to various phenomena, such as axotomy leading to the fracture of distal axons and myelin sheath leading to Wallerian degeneration. To repair the nervous system, Schwann cells (SCs) begin to proliferate, macrophages invade the distal nerve segment and phagocytize degradation substances. After the fragments were removed (Figure 1C), axons began regenerating.

**Figure 2:**
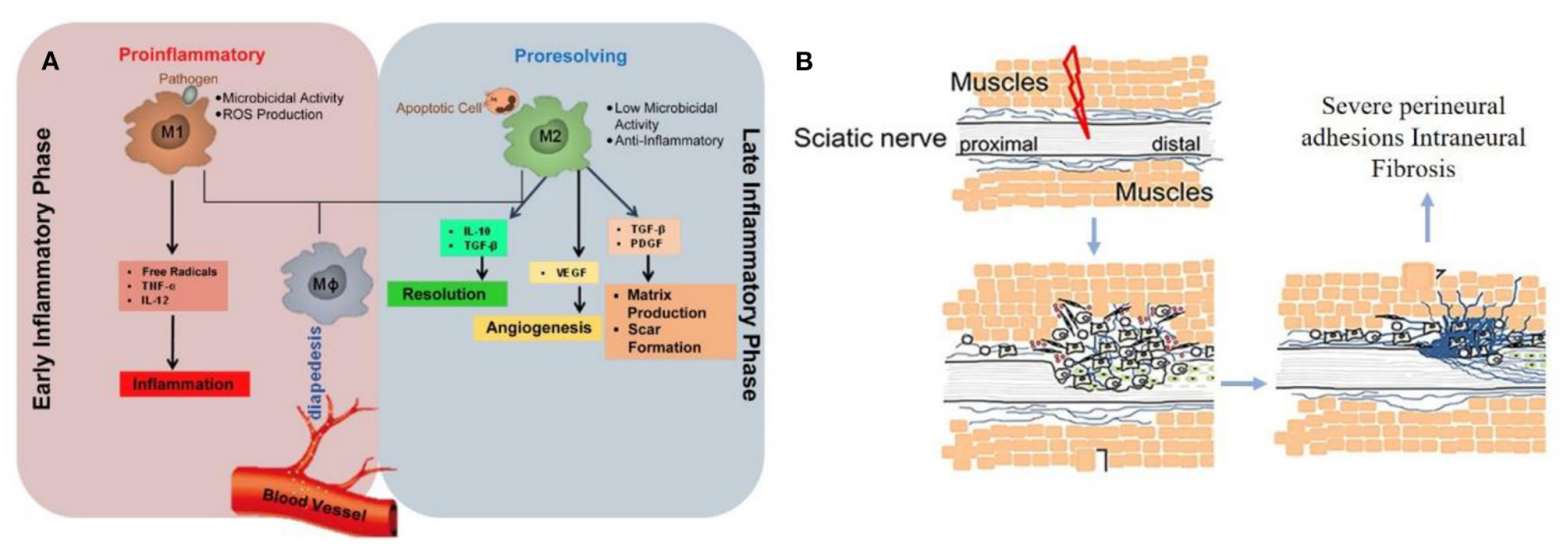
**(A)** Immunoregulatory properties of nerve repair macrophages reprinted (adapted) with permission from (22), Copyright (2015) Elsevier. **(B)** Excessive deposition of fibrous cells in the injured site leading to peripheral fibrosis reprinted (adapted) with permission from (18). Copyright (2019) Elsevier.

The perfect repair of damaged nerves inhibits the formation of inflammatory reactions, peripheral fibrosis, and scar tissue, thereby inhibiting or preventing neuroma formation. In the whole process of nerve repair, it was difficult to improve or block the occurrence of inflammation due to the significant differences between individuals. When the body was damaged, the immune system starts to collect granulocytes (neutrophils and mast cells) and agranulocytes (monocytes/macrophages and lymphocytes) to key positions in the first few hours (Figure 2A) (22). Effectively controlling neuroinflammatory diseases was a highly complex problem requiring specific intervention. In addition, fibrosis or scar tissue formation around the implant was another expected natural event after injury (Figure 2B) (18). However, the excessive deposition and subsequent remodeling of the extracellular matrix (ECM) will lead to permanent scars and eventually form a neuroma. Fibrosis can hinder regeneration in two ways: (i) Intraneural fibrosis will prevent axons from passing from proximal to distal segments; (ii) Extraneural fibrosis will limit the physiological movement of the nerves during exercise, leading to pain and functional limitations.

In terms of damage types, it is also necessary to distinguish between minor and long gaps. The small space (< 30 mm) for clinical treatment of nerve injury generally adopts autologous transplantation, allograft transplantation, and hollow lumen cannula. However, it is limited clinically due to insufficient autologous supply and allogeneic immune response. The injured nerve site must be protected as a guiding substrate for further repair. In addition, nutrients and oxygen diffuse to the regeneration site, so the hollow catheter is more demanding for nerve repair (23). Considering that most recoveries of long space injuries still have obstacles, this is because the distal stump is too far away from the proximal end, leading to the degradation of targeted organs. Worst of all, neuromas can also occur when the treatment of nerve injuries is ineffective. Therefore, these complex challenges could stimulate researchers to seek suitable new technologies to solve neuroma.

## 3. Emerging methods for treatment of traumatic neuroma

In clinic, the treatment of traumatic neuroma is mainly surgical treatment, but after the initial surgical treatment, up to 42% of patients may have persistent symptoms, which requires additional intervention (4). The determinants of secondary neuroma are complex, including patient symptoms, patient living environment, patient living habits, surgeon experience, and available reconstruction techniques. To avoid the occurrence of secondary neuroma and traumatic neuroma, it is necessary to develop new materials combined with clinical techniques. With the progress of materials science, stem cell biology, and bioengineering, new thinking models for treating diseases are increasingly taken into account in the scope of basic researches, transformations, and clinical applications (24). They can be used to explore the basic mechanisms of health and disease, such as *in vitro* tissue model drug screening and bridging or substitutes for trauma tissue. Although many “systemic drugs” have been developed clinically to guide the treatment of neuroma, it is now clear that locally mediated modified new materials can rapidly adjust the surrounding pathological environment to recommend the repair. In this review, we discussed how “local” biomaterials affect or treat neuromas, how stem cells secrete factors or self-regulate the surrounding environment of neuromas, and how human-computer interfaces treat neuromas.

### 3.1. Biomaterials for treatment of traumatic neuroma

In the early clinical stage, the neuroma is mainly treated by surgical resection, but it is easy to recur. In addition, nerve axons buried in adjacent tissues may lead to direct contraction or traction of nerves. Using nerve conduits to bridge or covering injured nerves is a promising method. Nerve grafts have become the gold standard for nerve reconstruction and neuroma prevention, while donor incidence rate and immunogenic host response remain challenges. With the rapid development of biotechnology and biomaterials, various biomaterials are being used to promote the functional recovery of injured nerves, prevent the occurrence of neuromas, and treat neuroma pain. The materials have been endowed with biological functions, from simple biopolymers to blends of bioactive substances and polymers. Additionally, the modifiability of materials endows them with multifunction, such as adjustable mechanical properties, degradability, good biocompatibility, convenience, and non-immunity. During the treatment period, biomaterials should also avoid swelling and not elicit inflammatory responses during degradation. Notably, the modified biomaterials should have the ability to maintain cell vitality and guide directional tissue growth.

**Figure 3:**
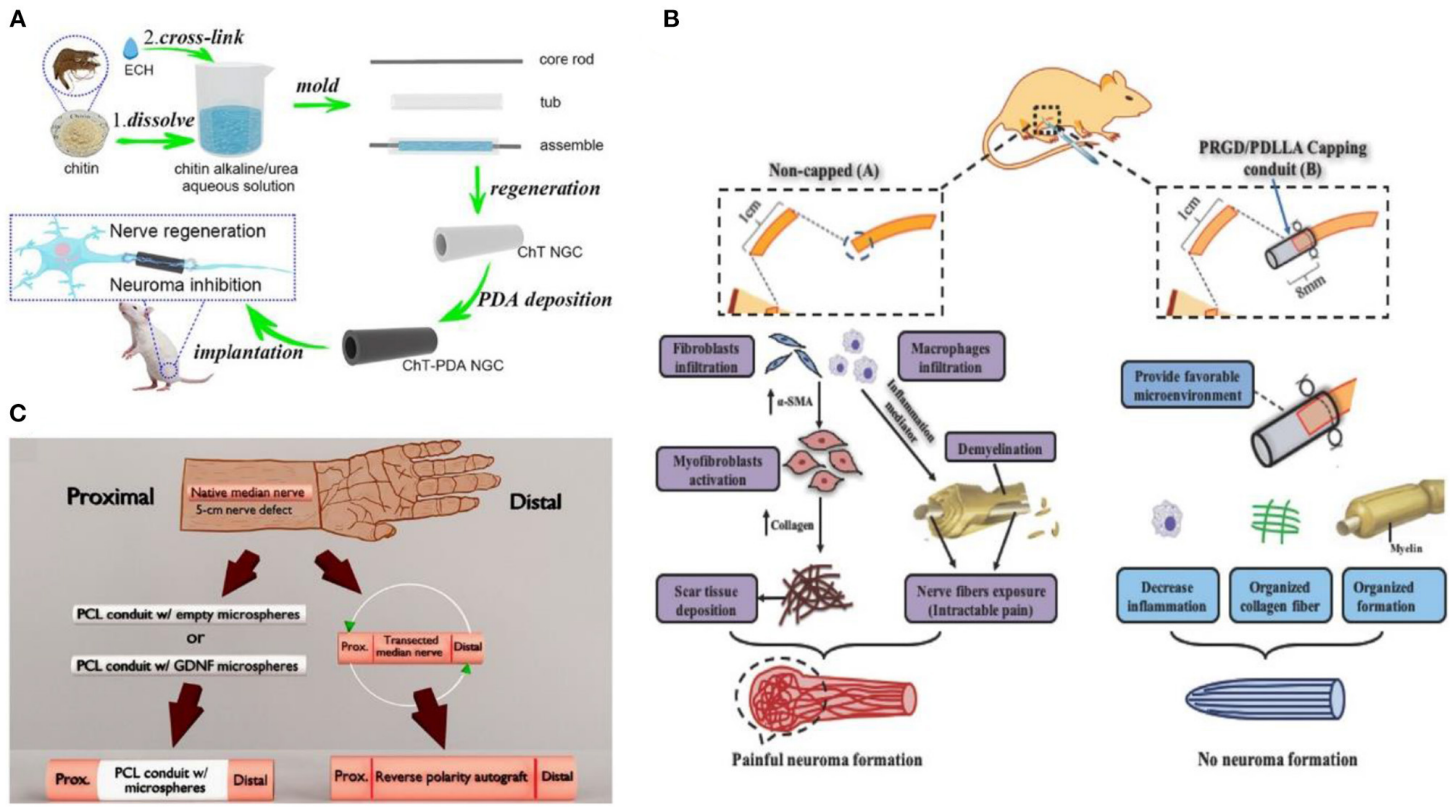
**(A)** Schematic diagram of nerve scaffold synthesis reprinted (adapted) with permission from (27), Copyright (2022) Elsevier. **(B)** Schematic of PRGD/PDLLA conduit in preventing traumatic neuroma reprinted (adapted) with permission from (28), Copyright (2018) Wiley. **(C)** Schematic diagram of nerve cannula design reprinted (adapted) with permission from (29), Copyright (2020) The American Association for the Advancement of Science.

In recent years, many researchers have been committed to replace promising alternative biomaterials to autologous or allogeneic nerve transplantation, and simulate the macro or micro ring structure of peripheral nerves to improve the therapeutic effect and inhibit the occurrence of neuroma (25, 26). Yang et al. (27) designed a biodegradable cannula based on chitin for peripheral nerve injury in long-segment defects. The hollow cannula was prepared through chitin dissolution, molding, and regeneration, and then the hollow cannula was immersed in the anti-inflammatory dopamine solution (Figure 3A). The deposited cannula shows excellent inhibition on nerve regeneration and neuroma. Yi et al. (28) synthesized that biocompatible poly(D, L-lactic acid)/arginyl glycyl aspartic acid (RGD peptide) modification of poly{(lactic acid)-co-[(glycolic acid)-alt-(L-lysine)]}(PRGD/PDLLA) nerve cannula (Figure 3B). The catheter inhibits inflammation by modifying RGD, providing an appropriate microenvironment for nerve endings recovery, and avoiding neuroma formation during nerve recovery. Given severe peripheral nerve injury, the possibility of neuroma formation caused by auto-transplantation was enhanced, and the sensory function of the donor site was lost. Fadia et al. (29) studied a biodegradable polycaprolactone catheter (Figure 3C). Glial cell-derived growth factor (GDNF) was wrapped in the catheter, which showed that the degradable nerve catheter effectively bridged the long peripheral nerve gap and prevented the occurrence of neuroma. In general, although a single cannula could promote nerve regeneration and reduce the occurrence of neuroma, the single cannula cannot fully meet clinical requirements.

**Figure 4:**
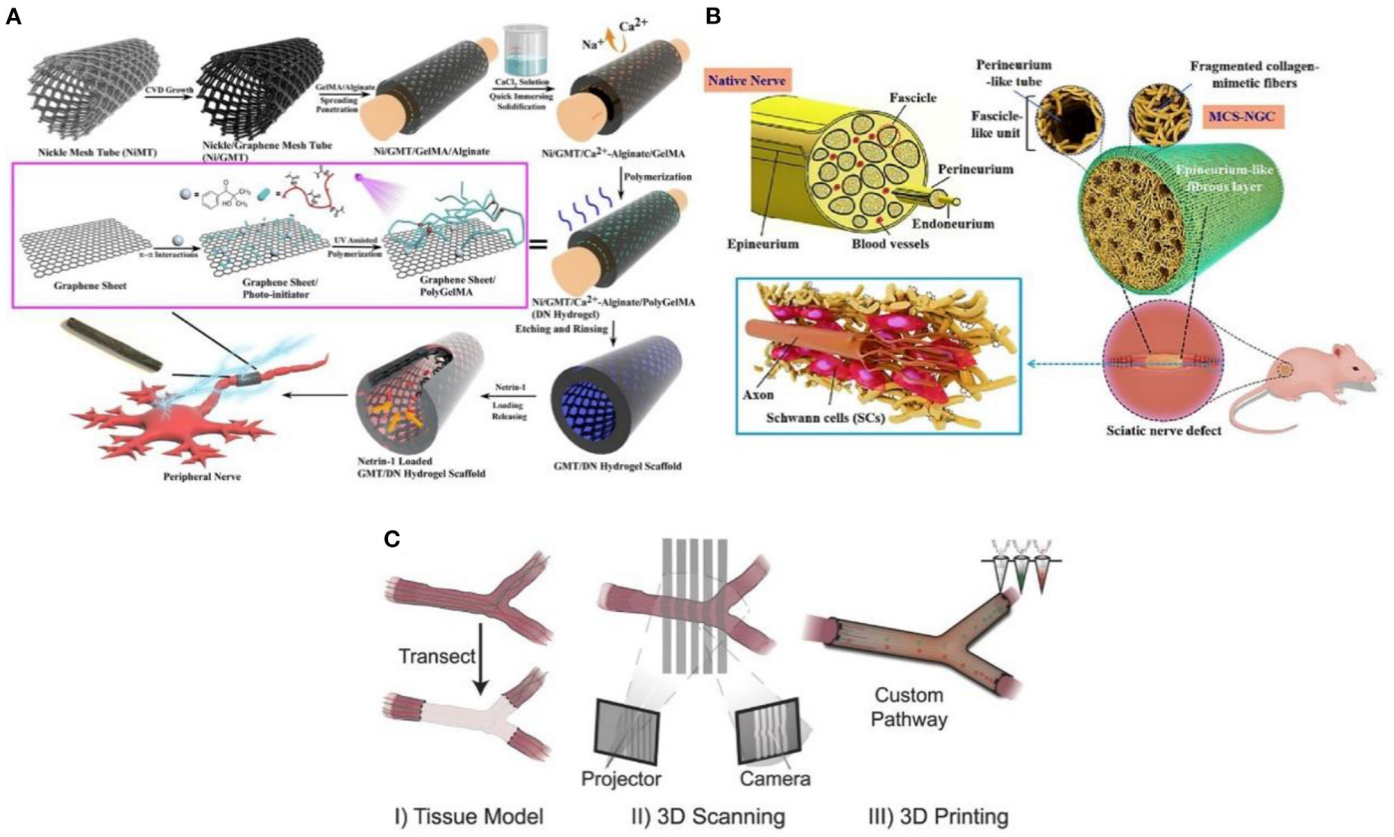
**(A)** The graphene mesh supported double network (DN) hydrogel scaffold is loaded with netrin-1 to form a neural sleeve reprinted (adapted) with permission from (35), Copyright (2021) American Chemical Society. **(B)** Multichannel nerve cannula reprinted (adapted) with permission from (37), Copyright (2020) Elsevier. **(C)** The 3D printing realizes personalized neural channel reprinted (adapted) with permission from (38), Copyright (2015) John Wiley and Sons.

The traditional casing manufacturing process is complex, time-consuming, and single in practicability. The emerging 3D printing technology is a revolutionary change for the effective prevention and treatment of neuroma (30). Additionally, 3D printing technology overcomes the permeability problem of traditional nerve cannulas. It can also simulate the structure and function of the peripheral nerve through biomaterials, biomolecules, growth factors, exosomes, and cells (31). Importantly, the size and morphology of the gap with nerve damage are diversified. For example, the axonal fibers of the same neuron are scattered to different target tissues, sometimes controlled by multiple nerves (32). However, 3D printing technology enables patients to create personalized cannulas to treat and prevent neuromas (33). The multifunctional 3D-printed nerve cannula actively promotes axon regeneration and nerve growth to avoid the occurrence of neuroma through various methods. For example, the implantation of growth factors (34), conductive biomaterials (35), and stem cells (36), lack in the neural environment into the neural cannula can realize the multi-function of the neural cannula. Huang et al. (35) fabricated a 3D graphene mesh tube (GMT) using nickel mesh as a template (Figure 4A). The graphene mesh can promote the proliferation of SCs and guide their alignments. Nerve stents can significantly promote the regeneration of peripheral nerves and the recovery of denervated muscles, which is even better than autologous transplantation.

Considering that the nerve is composed of many axons, the multi-lumen nerve cannula has better advantages than the single-lumen cannula in reducing the misorientation rate of the regenerative axons (39). For example, the large specific surface area in the multi-cavity catheter promotes cell adhesion and provides appropriate directional guidance for axon growth (40). Wang et al. (37) adopted a multi-scale strategy based on natural peripheral nerves' composition complexity and structure level. The tussah silk fibroin/poly (l-lactic acid-ε-Caprolactone)/graphene oxide (*Ap*F/PLCL/GO) nanofibers were used to prepare multicavity nerve conduits (Figure 4B). This customized 3D bionic nerve scaffold can improve the mechanical properties and almost completely degrade after 12 weeks *in vivo*. In addition to the single hole and porous nerve cannula, nerve damage may also occur at the bifurcation and taper. Based on this, Johnson et al. (38) produced a “dendritic” nerve cannula with silica gel as raw material through the designability and customization of 3D printing (Figure 4C), which was used to promote nerve regeneration and reduce the occurrence of neuroma. Importantly, 3D printing allows branch nerves to extend to targeted distal nerves. This mode is difficult to realize with traditional single-hole casing.

In general, the 3D printing stent has successfully regenerated complex nerve injuries, thus enhancing the functional recovery of the regenerative nerve. The 3D printing method used for nerve repair to prevent neuroma has the following potential advantages:

(1) Customize the geometry of the stent to match the inherent tissue anatomy (36);
(2) Combine the biological manufacturing method with the calculation modeling of design, analysis, and optimization;
(3) Use physical and biochemical functions of space control to enhance equipment performance.
(4) The personalized design endows the nerve scaffold with more clinical applicability.

### 3.2. Treatment of traumatic neuroma with stem cells

Considering that the formation of traumatic neuroma is due to the disorder accumulation of scar tissue, and the irregularity and immaturity of axon regeneration. The nerve tissue is a complex structure composed of multiple interacting cells. Due to the highly complex structure, it is difficult for researchers to assess the degree of nerve damage. According to different nerve injuries of patients, exploring new methods and strategies to develop more personalized treatment methods that are more suitable for clinical application is necessary. In this way, the formation of traumatic neuroma, especially to prevent the irregular axon the regeneration of neuroma and the disorder accumulation of scar tissue, could be thoroughly and effectively prevented (41).

After the damage to local cells and tissues, the body will repair the defects. Tissue repair is mainly a repair process through the division and proliferation of adjacent normal cells (16). Considering that the damaged organism causes tissue lesions, the key to repair is to restore the physiological function state. Furthermore, the maintenance and development of peripheral nerves depend on local signals between axons and SCs. The proliferation of SCs provides mechanical matrix and growth factors to promote axonal regeneration. In addition, factors secreted by SCs can activate signals for nerve repair. However, the procedure of extracting SCs is cumbersome. In addition, it is difficult to culture SCs *in vitro*. Stem cells are original undifferentiated cells with multipotent differentiation and self-replication ability, which are the original cells forming various tissues and organs (42). The extraction method of stem cells is simple, especially mesenchymal stem cells (MSCs). The pluripotent stem cells can activate the “self-healing function” of the human body itself, supplement and regulate the diseased cells, activate the cell function, improve the activity and quality of cells, and finally restore the normal physiological function of cells (43).

**Figure 5:**
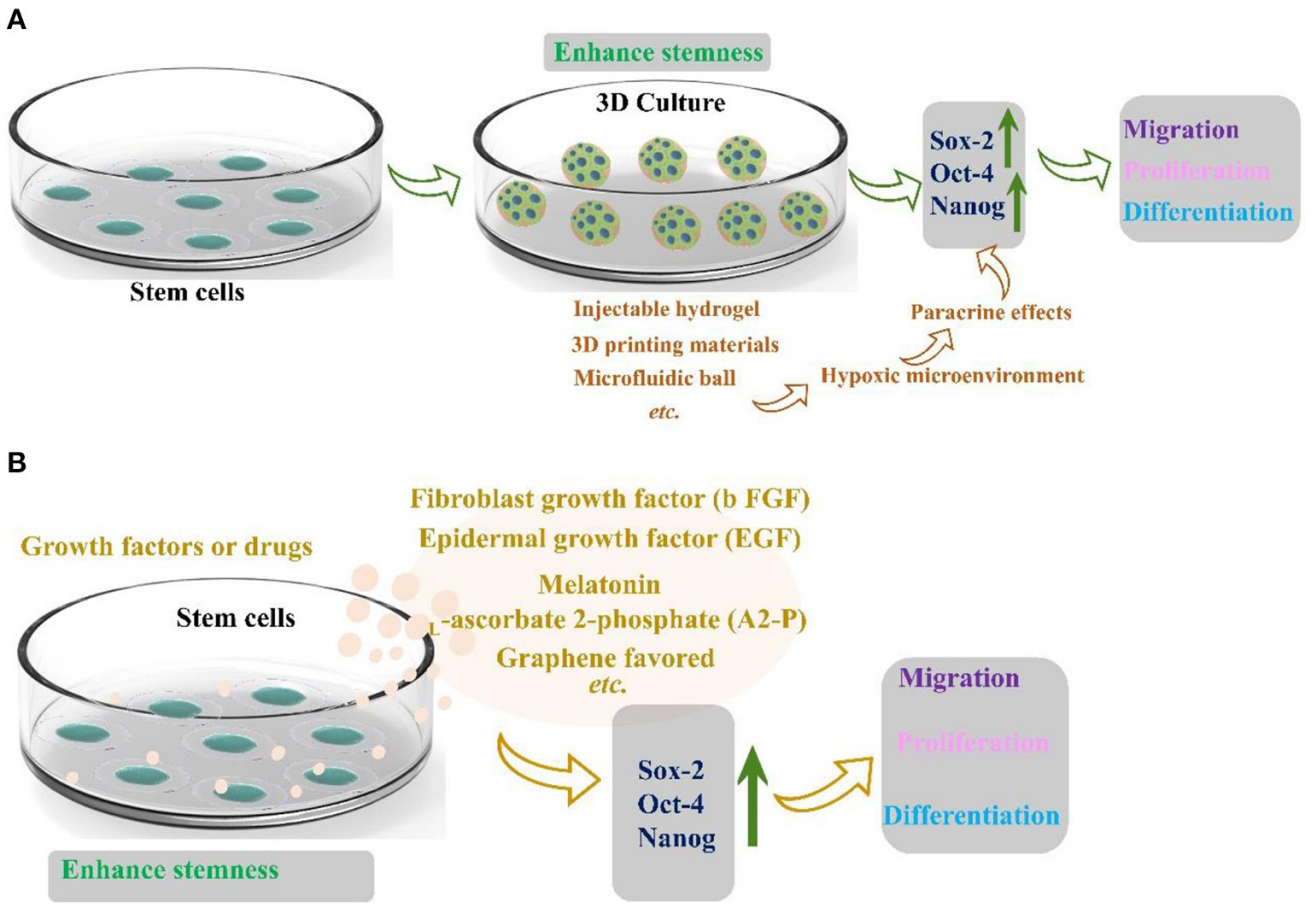
**(A)** Three-dimensional structure maintains the stemness of stem cells reprinted (adapted) with permission from (47), Copyright (2022) American Chemical Society. **(B)** Growth factors and drugs maintain the stemness of stem cells (47).

In 2017, Kizilay et al. (44) found that bone marrow mesenchymal stem cells are a promising regeneration trigger following axonal and nerve damage. Masgutov et al. (45) prepared a method to transport MSCs to the traumatic injury area using fibrin glue. Collagen scaffold immobilizes cells and also provides extracellular matrix support. The researchers thoroughly used the sciatic nerve injury model to demonstrate that sensory nerves protect neurons and stimulate axon growth and myelination. The phenotype of sensory neurons was also improved after trauma. Significantly, MSCs promote neuro angiogenesis and motor function recovery. Masgutov et al. (46) studied the effect on rat sciatic nerve regeneration after trauma by transplanting human-derived stem cells (hADSCs). His team built a complete sciatic nerve transection model. After 2 months of repair, the results showed that hADSC promoted the survival of neurons in the spinal ganglia, announced axon repair, and stimulated peripheral nerve regeneration (46). Although stem cells are rich in resources and advanced in extraction technology, stem cells gradually lose the ability of directional differentiation after long-term *in vitro* culture. The current method was to encapsulate stem cells in three-dimensional (3D) spheres to maintain and promote the stemness of stem cells, which can play a paracrine effect in three-dimensional space due to hypoxia (Figure 5A) (47). Furthermore, stemness maintenance could also be realized by adding growth factors or drugs (Figure 5B) (47). Otherwise, bone marrow stem cells are the first seed cells to conduct clinical application research. Because of their self-origin and harvest, they will cause less damage to the body compared with other tissues. However, the quantity and quality of bone marrow stem cells are closely related to the age and quality of individuals. With the increase of age, the quantity and quality of bone marrow stem cells will decrease dramatically. The incidence rate of some diseases will increase sharply with age. For older patients, there is a practical problem that it is challenging to use autologous bone marrow stem cells to transplant and repair related diseases. Therefore, the critical issue to be solved in regenerative medicine today is finding stem cell sources with rich sources, convenient harvest, low immunity, and multi-differentiation potential. The role of stem cells in neural repair has many beneficial and exciting things to be developed by researchers. In the future, researchers should maximize the advantages of stem cells and gather them into neural repair projects to make them more suitable for clinical application.

### 3.3. Treatment of traumatic neuroma with human machine interface

**Figure 6:**
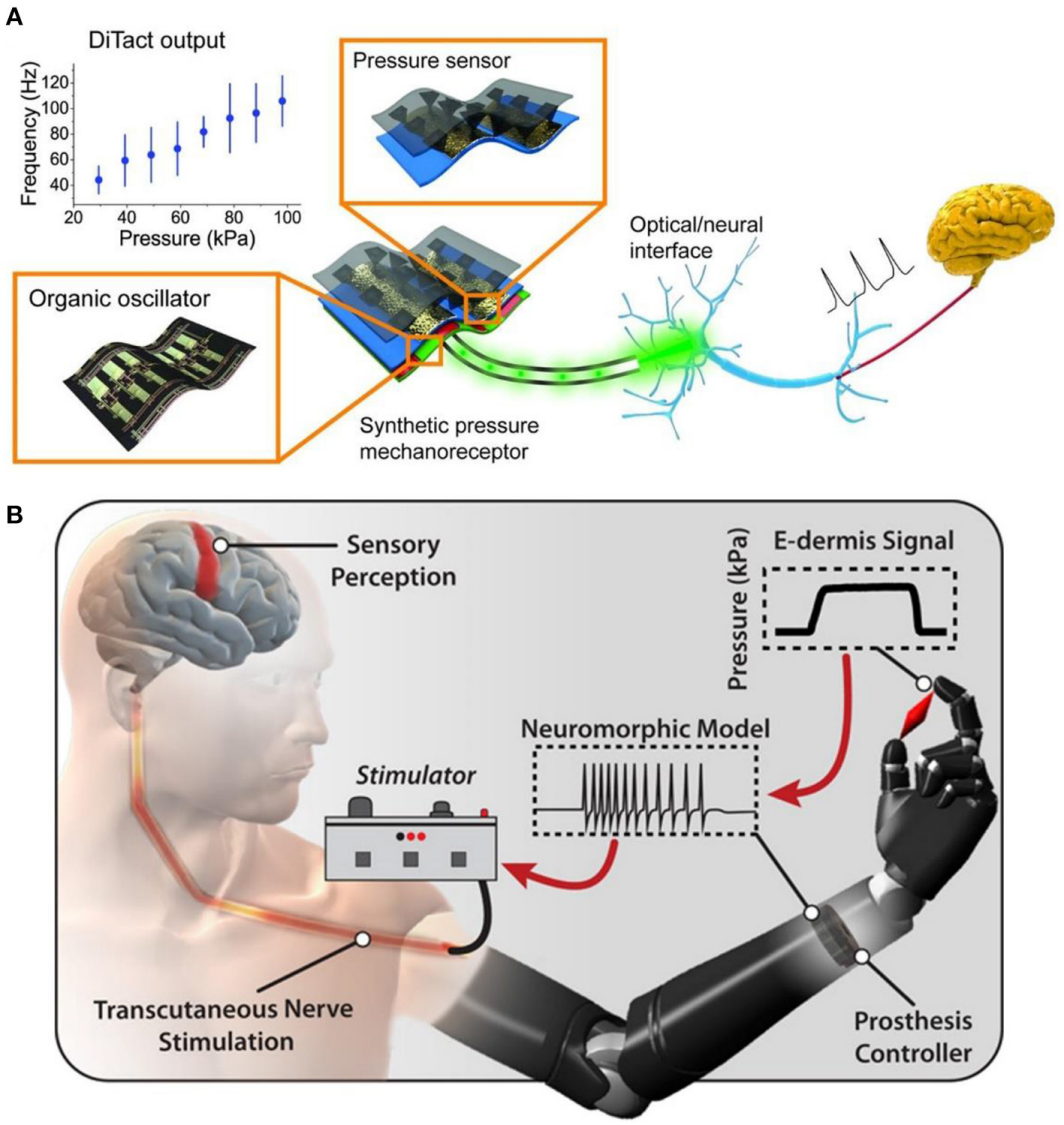
**(A)** A power efficient skin-inspired mechanoreceptor reprinted (adapted) with permission from (52), Copyright (2015) The American Association for the Advancement of Science. **(B)** A neuromorphic interface producing receptor like spiking neural activity for tactile stimuli feedback reprinted (adapted) with permission from (53), Copyright (2018) The American Association for the Advancement of Science.

In recent years, soft robots, nanorobots, and flexible electronic interfaces have attracted great attention due to their good biocompatibility, low cost, high security, versatility, and adaptability (48). Medical performance robot is a treasure for non-drug treatment of diseases, especially the man-machine interface equipment used for artificial limb or nerve repair, which could significantly improve the quality of life of injured patients (49). According to the United States Centers for Disease Control data, 13.7% of Americans with disabilities have mobility disorders, and 10.8% have cognitive disorders (50). These barriers could be alleviated through medical robots and new methods. In the twentifirst century, 3D printing technology will gradually replace complex, heavy, and insensitive metal robot brackets to achieve flexibility, portability, and safety. At the end of the twenteeth century, researchers found that rehabilitation training, brain function, acting ability, and neural function loss were significantly improved after a long period (51). The nerve was a complex sensory and response signal processing system that senses subtle changes in the environment. Once the nerve is damaged and the signal transmission is interrupted, a slight carelessness in the follow-up treatment and repair may evolve into a neuroma. However, due to the lack of interface between mechanical parts and the human body, the external signal of the robot cannot be transferred to the nerve as the skin communication path (Figure 6A) (52). Although *in vitro* and *in vivo* artificial sensory nerves could communicate with cells through optical methods, they have not yet reached the link with a single neuron. Based on the concept of electronic skin sensing (54), if tiny sensory devices are transplanted and integrated into the “machine with the artificial interface,” they could be used for nerve repair to prevent neuroma.

The pain of patients will be caused during and after the repair of nerve injury. Especially after amputation, neuroma caused by neuropathy affects the normal quality of life of patients. Amputees complete a series of activities in life by using artificial limbs. To prevent further injury and pain in amputees, Osborn et al. (53) developed a multi-layer electronic dermis (e-dermis) and a neuro morphological interface (Figure 6B), which proved the ability of the prosthesis and the users to distinguish safe (harmless) and painful (harmful) tactile sensation during grasping. They used the prosthesis reflex (simulated as a multi-synaptic retraction reflex) to respond appropriately. With the advent of the digital era, in the case of biocompatibility, the search for functional materials of the human-computer interface can gradually reduce or prevent various injuries in patients and promote nerve repair. The researchers sought to combine biomaterials that could transmit signals with “artificial robots” to reduce neural pain. These two methods are the mainstream research directions of the human-computer interface. In the increasingly developing spiritual culture, researchers should strive to improve patients' quality of life.

The main challenge of the human-computer interface mainly comes from materials. The existing machines mostly use traditional materials, such as metal, silicon, glass, ceramics, and plastics. However, these conventional materials' hard, dry and non-biological characteristics are essentially contradictory to biological tissues' soft, wet, and living characteristics (55). Furthermore, the existing human-computer interface materials only change the structure design. They do not modify the inherent characteristics of the materials, which may still hinder their communication and interaction with biological tissues. In addition, biological tissues of the body often identify these materials as foreign bodies, which will seriously damage the long-term reliability and effectiveness of communication and interaction between people and machines (56). Notably, developing new materials also faces many scientific, engineering, and transformation challenges. Researchers should devote themselves to the development of biological materials for the combination of natural systems and abiotic systems to better integrate humans and computers. Based on the above characteristics, we summarized the new development direction of the human-computer interface.

- Considering the softness and wetness of biological tissue, the hydrogel is a promising candidate material for bioengineering;
- In addition to considering the design of materials, researchers should also endow materials with multi-function to better bionic tissue;
- Human-computer interface materials should be combined with more bionics and tissues to improve long-term safety and accuracy.

## 4. Clinical trials to evaluate the effectiveness and safety of new technologies and methods

Clinical trial refers to any systematic study of drugs in humans (patients or healthy volunteers) to confirm or reveal the effects, adverse reactions and/or absorption, distribution, metabolism, and excretion of the test drugs (57). The purpose is to determine the efficacy and safety of the test drugs (58). Considering the protection of patients' interests, a new drug must undergo clinical trials before entering clinical practice to obtain pharmacologically (effect) and toxicological (adverse reaction) data and effective rate and survival period data (57–60). Therefore, it is necessary to conduct clinical trials on the effectiveness and safety of new technologies and methods. We summarize the clinical trials of some existing new materials as follows (Table 2).

**Table 2:** Summary of clinical trials of existing materials.

**Materials**	**Therapeutic pathology**	**Clinical evidence**	**Ref**
Chitosan nerve tube	Treatment of permanent loss of sensitivity and painful neuroma	Therapeutic, I.	(57)
Chitosan membrane (ChiMe)	The neurovascular bundles (NVBs) after nerve-sparing robot-assisted radical prostatectomy (NS-RARP)	Therapeutic, II.	(58)
PGLA [poly(glycolide-co-L-lactide)] nerve conduit	Treatment of nerve injury and nerve repair and regeneration	Therapeutic, I.	(59)
Glial cell line-derived neurotrophic factor (GDNF)	Treatment of Amyotrophic lateral sclerosis (ALS)	Therapeutic, phase 1/2a trial	(60)

## 5. Conclusion

To sum up, many research results have sound effects on the prevention and treatment of neuromas, such as reducing the incidence rate and the pain of patients. In the future, it should be easier to meet the needs of treating traumatic neuroma based on the advantages of existing treatment methods and combined with existing science and technology. This review focuses on preventing and treating neuroma with biomaterials, stem cells, and human-computer interfaces. Although these scientific researches are only at the research stage, they are also a big step toward clinical application. From the perspective of medical materials, the effectiveness and safety of all materials are the core issues in the treatment and prevention of neuroma. An effective design will better solve the complex problems in the treatment and prevention of neuroma, for example, how the existing human-computer interface metal materials deal with the humid environment of biology. Regarding safety, how to effectively avoid adverse reactions, such as inflammation, metabolism, toxicity, and antigen reaction in tissues. Furthermore, expensive materials will also bring financial pressure on patients. In future clinical work, the treatment and prevention of neuroma is still a scientific problem. Interdisciplinary methods such as biomaterials, 3D printing technology, stem cells, and human-computer interface can assist, intervene, prevent, and treat neuromas. Therefore, the interdisciplinary combination of medicine and biomaterials must be an essential way to treat neuroma and nerve repair in the future.

## Author contributions

LZ: writing and editing the manuscript. TH and WZ: investigation and visualization. NH: supervision and writing-review and editing. YW and PZ: supervision, conceptualization, writing-review and editing, and funding acquisition. All authors contributed to the article and approved the submitted version.

## References

[1] Devor M Wall PD. Type of sensory nerve fibre sprouting to form a neuroma. Nature. (1976) 262:705–8. 10.1038/262705a0 958442

[2] Foltán R Klíma K Špačková J Šedý J. Mechanism of traumatic neuroma development. Med Hypotheses. (2008) 71:572–6. 10.1016/j.mehy.2008.05.010 18599222

[3] Hooper RC Cederna PS Brown DL Haase SC Waljee JF Egeland BM. Regenerative peripheral nerve interfaces for the management of symptomatic hand and digital neuromas. Plastic Reconstr Surg Global Open. (2020) 8:e2792. 10.1097/GOX.0000000000002792 32766027 PMC7339232

[4] Wolvetang NHA Lans J Verhiel SHWL Notermans BJW Chen NC Eberlin KR. Surgery for symptomatic neuroma: anatomic distribution and predictors of secondary surgery. Plast. Reconstr. Surg. (2019) 143:664. 10.1097/PRS.0000000000005664 30907815

[5] Elliot D. Surgical management of painful peripheral nerves. Clin Plastic Surg. (2014) 41:589–613. 10.1016/j.cps.2014.03.004 24996473

[6] Stokvis A van der Avoort DJC van Neck JW Hovius SER Coert JH. Surgical management of neuroma pain: a prospective follow-up study. Pain. (2010) 151:862–9. 10.1016/j.pain.2010.09.032 20974520

[7] Vaegter HB Andersen PG Madsen MF Handberg G Enggaard TP. Prevalence of neuropathic pain according to the IASP grading system in patients with chronic non-malignant pain. Pain Med. (2014) 15:120–7. 10.1111/pme.12273 24165161

[8] Finnerup NB Kuner R Jensen TS. Neuropathic pain: From mechanisms to treatment. Physiol Rev. (2020). 10.1152/physrev.00045.2019 32584191

[9] Penna A Konstantatos AH Cranwell W Paul E Bruscino-Raiola F-R. Incidence and associations of painful neuroma in a contemporary cohort of lower-limb amputees. Aust N Z J Surg. (2018) 88:491–6. 10.1111/ans.14293 29654613

[10] Kryger GS Kryger Z Zhang F Shelton DL Lineaweaver WC Buncke HJ. Nerve growth factor inhibition prevents traumatic neuroma formation in the rat. J Hand Surg Am. (2001) 26:635–44. 10.1053/jhsu.2001.26035 11466637

[11] Yao C Zhou X Weng W Poonit K Sun C Yan H. Aligned nanofiber nerve conduits inhibit alpha smooth muscle actin expression and collagen proliferation by suppressing TGF-β1/SMAD signaling in traumatic neuromas. Exp Ther Med. (1414) 2021:22. 10.3892/etm.2021.10850 34676007 PMC8527191

[12] Kubiak CA Kung TA Brown DL Cederna PS Kemp SWP. State-of-the-art techniques in treating peripheral nerve injury. Plast. Reconstr. Surg. (2018) 141:720–13. 10.1097/prs.0000000000004121 29140901

[13] Kubiak CA Kemp SWP Cederna PS. Regenerative peripheral nerve interface for management of postamputation neuroma. JAMA Surg. (2018) 153:681–2. 10.1001/jamasurg.2018.0864 29847613

[14] Woo SL Kung TA Brown DL Leonard JA Kelly BM Cederna PS. Regenerative peripheral nerve interfaces for the treatment of postamputation neuroma pain: a pilot study. Plastic Reconst Surg Global Open. (2016) 4:1038. 10.1097/GOX.0000000000001038 28293490 PMC5222635

[15] He FL Qiu S Zou JL Gu FB Yao Z Tu ZH. Covering the proximal nerve stump with chondroitin sulfate proteoglycans prevents traumatic painful neuroma formation by blocking axon regeneration after neurotomy in Sprague Dawley rats. J Neurosurg. (2020) 134:1599–609. 10.3171/2020.3.JNS193202 32470939

[16] Zhou L Pi W Cheng S Gu Z Zhang K Min T. Multifunctional DNA hydrogels with hydrocolloid-cotton structure for regeneration of diabetic infectious wounds. Adv Funct Mater. (2021) 31:2106167. 10.1002/adfm.202106167

[17] Zhou J Li J Du X Xu B. Supramolecular biofunctional materials. Biomaterials. (2017) 129:1–27. 10.1016/j.biomaterials.2017.03.014 28319779 PMC5470592

[18] Carvalho CR Silva-Correia J Oliveira JM Reis RL. Nanotechnology in peripheral nerve repair and reconstruction. Adv Drug Delivery Rev. (2019) 148:308–43. 10.1016/j.addr.2019.01.006 30639255

[19] Grinsell D Keating CP. Peripheral nerve reconstruction after injury: a review of clinical and experimental therapies. BioMed Res. Int. (2014) 2014:698256. 10.1155/2014/698256 25276813 PMC4167952

[20] Allodi I Udina E Navarro X. Specificity of peripheral nerve regeneration: Interactions at the axon level. Prog Neurobiol. (2012) 98:16–37. 10.1016/j.pneurobio.2012.05.005 22609046

[21] Gu X Ding F Williams DF. Neural tissue engineering options for peripheral nerve regeneration. Biomaterials. (2014) 35:6143–56. 10.1016/j.biomaterials.2014.04.064 24818883

[22] Das A Sinha M Datta S Abas M Chaffee S Sen CK. Monocyte and macrophage plasticity in tissue repair and regeneration. Am J Pathol. (2015) 185:2596–606. 10.1016/j.ajpath.2015.06.001 26118749 PMC4607753

[23] Stang F Keilhoff G Fansa H. Biocompatibility of different nerve tubes. Materials. (2009) 2:1480–507. 10.3390/ma2041480

[24] Blache U Stevens MM Gentleman E. Harnessing the secreted extracellular matrix to engineer tissues. Nat Biomed Eng. (2020) 4:357–63. 10.1038/s41551-019-0500-6 31974449 PMC7180075

[25] Qian Y Zhao X Han Q Chen W Li H Yuan W. An integrated multi-layer 3D-fabrication of PDA/RGD coated graphene loaded PCL nanoscaffold for peripheral nerve restoration. Nat Commun. (2018) 9:323. 10.1038/s41467-017-02598-7 29358641 PMC5778129

[26] Qian Y Song J Zheng W Zhao X Ouyang Y Yuan W. 3D manufacture of gold nanocomposite channels facilitates neural differentiation and regeneration. Adv Funct Mater. (2018) 28:1707077. 10.1002/adfm.201707077

[27] Yang X Huang L Yi X Huang S Duan B Yu A. Multifunctional chitin-based hollow nerve conduit for peripheral nerve regeneration and neuroma inhibition. Carbohydr Polym. (2022) 289:119443. 10.1016/j.carbpol.2022.119443 35483856

[28] Yi J Jiang N Li B Yan Q Qiu T Swaminatha Iyer K. Painful terminal neuroma prevention by capping PRGD/PDLLA conduit in rat sciatic nerves. Adv Sci. (2018) 5:1700876. 10.1002/advs.201700876 29938170 PMC6010769

[29] Fadia NB Bliley JM DiBernardo GA Crammond DJ Schilling BK Sivak WN. Long-gap peripheral nerve repair through sustained release of a neurotrophic factor in nonhuman primates. Sci. Transl. Med. (2020) 12:eaav7753. 10.1126/scitranslmed.aav7753 31969488

[30] Jakus AE Secor EB Rutz AL Jordan SW Hersam MC Shah RN. Three-dimensional printing of high-content graphene scaffolds for electronic and biomedical applications. ACS Nano. (2015) 9:4636–48. 10.1021/acsnano.5b01179 25858670

[31] Liu Y Hsu Sh. Biomaterials and neural regeneration. Neural Regener. Res. (2020) 15:1243. 10.4103/1673-5374.274331 31960803 PMC7047791

[32] Lee SJ Zhu W Heyburn L Nowicki M Harris B Zhang LG. Development of novel 3-d printed scaffolds with core-shell nanoparticles for nerve regeneration. IEEE Trans Biomed Eng. (2017) 64:408–18. 10.1109/TBME.2016.2558493 28113194

[33] Liu K Yan L Li R Song Z Ding J Liu B. 3D Printed personalized nerve guide conduits for precision repair of peripheral nerve defects. Adv Sci. (2022) 9:2103875. 10.1002/advs.202103875 35182046 PMC9036027

[34] Choi YJ Jun YJ Kim DY Yi HG Chae SH Kang J. A 3D cell printed muscle construct with tissue-derived bioink for the treatment of volumetric muscle loss. Biomaterials. (2019) 206:160–9. 10.1016/j.biomaterials.2019.03.036 30939408

[35] Huang Q Cai Y Zhang X Liu J Liu Z Li B. Aligned graphene mesh-supported double network natural hydrogel conduit loaded with netrin-1 for peripheral nerve regeneration. ACS Appl Mater Interfaces. (2021) 13:112–22. 10.1021/acsami.0c16391 33397079

[36] Lind JU Busbee TA Valentine AD Pasqualini FS Yuan H Yadid M. Instrumented cardiac microphysiological devices via multimaterial three-dimensional printing. Nat Mater. (2017) 16:303–8. 10.1038/nmat4782 27775708 PMC5321777

[37] Wang J Cheng Y Wang H Wang Y Zhang K Fan C. Biomimetic and hierarchical nerve conduits from multifunctional nanofibers for guided peripheral nerve regeneration. Acta Biomater. (2020) 117:180–91. 10.1016/j.actbio.2020.09.037 33007489

[38] Johnson BN Lancaster KZ Zhen G He J Gupta MK Kong YL. 3D printed anatomical nerve regeneration pathways. Adv Funct Mater. (2015) 25:6205–17. 10.1002/adfm.201501760 26924958 PMC4765385

[39] Zeng CG Xiong Y Xie G Dong P Quan D. Fabrication and evaluation of PLLA multichannel conduits with nanofibrous microstructure for the differentiation of NSCs in vitro. Tissue Eng, Part A. (2014) 20:1038–48. 10.1089/ten.tea.2013.0277 24138342 PMC3938950

[40] Bender MD Bennett JM Waddell RL Doctor JS Marra KG. Multi-channeled biodegradable polymer/CultiSpher composite nerve guides. Biomaterials. (2004) 25:1269–78. 10.1016/j.biomaterials.2003.08.046 14643601

[41] Mandrycky C Wang Z Kim K Kim DH. 3D bioprinting for engineering complex tissues. Biotechnol Adv. (2016) 4:422–34. 10.1016/j.biotechadv.2015.12.011 26724184 PMC4879088

[42] Zhang RC Du WQ Zhang JY Yu SX Lu FZ Ding HM Cheng YB. Mesenchymal stem cell treatment for peripheral nerve injury: a narrative review. Neural Regener Res. (2021) 16:2170–6. 10.4103/1673-5374.310941 33818489 PMC8354135

[43] Zhou L Zeng Z Liu S Min T Zhang W Bian X Du H Zhang P Wen Y. Multifunctional DNA hydrogel enhances stemness of adipose-derived stem cells to activate immune pathways for guidance burn wound regeneration. Adv. Funct. Mater. (2022) 33:2207466. 10.1002/adfm.202207466

[44] Kizilay Z Aktas S Kahraman Cetin N Bakay Ilhan D Ersoy G Erken HA. Effect of systemic application of bone marrow-derived mesenchymal stem cells on healing of peripheral nerve injury in an experimental sciatic nerve injury model. Turk Neurosurg. (2017) 28:654–62. 10.5137/1019-5149.JTN.20811-17.1 28944943

[45] Masgutov R Masgutova G Mullakhmetova A Zhuravleva M Shulman A Rogozhin A. Adipose-derived mesenchymal stem cells applied in fibrin glue stimulate peripheral nerve regeneration. Front Med. (2019) 6:68. 10.3389/fmed.2019.00068 31024916 PMC6465797

[46] Masgutov RF Masgutova GA Zhuravleva MN Salafutdinov II Mukhametshina RT Mukhamedshina YO. Human adipose-derived stem cells stimulate neuroregeneration. Clin Exp Med. (2016) 16:451–61. 10.1007/s10238-015-0364-3 26047869

[47] Zhou L Min T Bian X Dong Y Zhang P Wen Y. Rational design of intelligent and multifunctional dressing to promote acute/chronic wound healing. ACS Appl Bio Mater. (2022) 5:4055–85. 10.1021/acsabm.2c00500 35980356

[48] Krebs HI Dipietro L Levy-Tzedek S Fasoli SE Rykman-Berland A Zipse J. A paradigm shift for rehabilitation robotics. IEEE Engineering in Medicine and Biology Magazine. (2008) 27:61–70. 10.1109/MEMB.2008.91949819004697

[49] Heng W Solomon S Gao W. Flexible electronics and devices as human–machine interfaces for medical robotics. Adv Mater. (2022) 34:2107902. 10.1002/adma.202107902 34897836 PMC9035141

[50] Laut J Porfiri M Raghavan P. The present and future of robotic technology in rehabilitation. Curr Phy Med Rehab Rep. (2016) 4:312–9. 10.1007/s40141-016-0139-0 28603663 PMC5461931

[51] Ashley MJ. Repairing the injured brain: why proper rehabilitation is essential to recovering function. Cerebrum. (2012) 212:8. Available online at: http://dana.org/news/cerebrum/detail.aspx?id=39258 23447794 PMC3574768

[52] Tee BC Chortos A Berndt A Nguyen AK Tom A McGuire A. A skin-inspired organic digital mechanoreceptor. Science (New York, NY). (2015) 350:313–6. 10.1126/science.aaa9306 26472906

[53] Osborn LE Dragomir A Betthauser JL Hunt CL Nguyen HH Kaliki RR Thakor NV. Prosthesis with neuromorphic multilayered e-dermis perceives touch and pain. Sci. Rob. (2018) 3:eaat3818. 10.1126/scirobotics.aat3818 32123782 PMC7051004

[54] Lai YC Ye BW Lu CF Chen CT Jao MH Su WF. Extraordinarily Sensitive and Low-Voltage Operational Cloth-Based Electronic Skin for Wearable Sensing and Multifunctional Integration Uses: A Tactile-Induced Insulating-to-Conducting Transition. Adv Funct Mater. (2016) 26:1286–95. 10.1002/adfm.201503606

[55] Yuk H Wu JJ Zhao XH. Hydrogel interfaces for merging humans and machines. Nat Rev Mater. (2022) 7:935–52. 10.1038/s41578-022-00483-4

[56] Lee GH Moon H Kim H Lee GH Kwon W Yoo S. Multifunctional materials for implantable and wearable photonic healthcare devices. Nat Rev Mater. (2020) 5:149–65. 10.1038/s41578-019-0167-3 32728478 PMC7388681

[57] Neubrech F Sauerbier M Moll W Seegmüller J Heider S Harhaus L. Enhancing the outcome of traumatic sensory nerve lesions of the hand by additional use of a chitosan nerve tube in primary nerve repair: a randomized controlled bicentric trial. Plast Reconstr Surg. (2018) 142:415–24. 10.1097/PRS.0000000000004574 30045179

[58] Porpiglia F Bertolo R Fiori C Manfredi M Cillis SD Geuna S. Chitosan membranes applied on the prostatic neurovascular bundles after nerve-sparing robot-assisted radical prostatectomy: a phase II study. BJU Int. (2017) 121:472–8. 10.1111/bju.13959 28710845

[59] Gao D Jiang JJ Gu SH Lu JZ Xu L. Morphological detection and functional assessment of regenerated nerve after neural prosthesis with a PGLA nerve conduit. Sci Rep. (46403) 2017:7. 10.1038/srep46403 28406160 PMC5390286

[60] Baloh RH Johnson JP Avalos P Allred P Svendsen S Gowing G. Svendsen Transplantation of human neural progenitor cells secreting GDNF into the spinal cord of patients with ALS: a phase 1/2a trial. Nat Med. (2022) 28:1813–22. 10.1038/s41591-022-01956-3 36064599 PMC9499868

